# Mr. Herbert Nelson Cappe

**Published:** 1910-05-07

**Authors:** 


The death is announced of Mr. Herbert Nelson Cappe, senior medical officer, Surrey County Asylum, Brookwood, at the age of forty-four. Mr. Cappe, who died of septicæmia, studied at University College, London, and took his diploma in 1888. He was a member of the Medical Psychological Society.

